# 高危细胞遗传学异常在多发性骨髓瘤中的预后价值

**DOI:** 10.3760/cma.j.cn121090-20250120-00035

**Published:** 2025-10

**Authors:** 旭星 沈, 佳培 郁, 睿 郭, 影 徐, 媛媛 金, 青林 史, 丽娟 陈

**Affiliations:** 南京医科大学第一附属医院，江苏省人民医院血液科，南京 210029 Department of Hematology, the First Affiliated Hospital of Nanjing Medical University, Jiangsu Province Hospital, Nanjing 210029, China

## Abstract

回顾性分析南京医科大学第一附属医院2016年12月至2024年12月收治的465例初治多发性骨髓瘤（NDMM）患者的临床资料，比较mSMART 3.0和mSMART 4.0风险分层系统下高危细胞遗传学异常（HRCA）在NDMM患者中的预后价值。结果显示，两种分层体系中，高危患者的预后均差于标危患者，且携带HRCA数量越多，患者预后越差。mSMART 4.0分层体系综合考虑了不同细胞遗传学异常共存的情况，对高危细胞遗传学的定义较mSMART 3.0分层体系更精准，区分不同细胞遗传学异常风险的能力更强。

## Abstract (en)

To retrospectively analyze the clinical data of 465 newly diagnosed patients with multiple myeloma（NDMM）admitted to the First Affiliated Hospital of Nanjing Medical University from December 2016 to December 2024, and compare the prognostic value of high-risk cytogenetic abnormalities（HRCAs）in NDMM patients under mSMART 3.0 and mSMART 4.0 risk stratification systems. The results showed that in both stratification systems, the prognosis of high-risk patients was worse than that of standard-risk patients. Moreover, a higher number of HRCAs was associated with a worse prognosis. The mSMART 4.0 system, which considers the coexistence of various cytogenetic abnormalities, provides a more precise definition of HRCA than mSMART 3.0. It demonstrates a superior ability to differentiate between different categories of cytogenetic risk.

多发性骨髓瘤（MM）细胞遗传学的多样性及复杂性是MM患者生存结局存在显著异质性的主要原因。检测技术的进步及对MM发病机制的深入研究使细胞遗传学异常成为MM预后危险分层的重要组成部分。随着对遗传学风险的深入认识及新药的涌现，不同危险分层指导下的个体化精准治疗有望改善患者的预后。Mayo骨髓瘤分层及风险调整治疗（Mayo Stratification of Myeloma and Risk-adapted Therapy，mSMART）分层系统是美国梅奥诊所基于各种危险因素构建的MM预后分层体系。近日，mSMART更新了4.0版本，对高危细胞遗传学异常（HRCA）的定义有较大调整。本研究回顾性分析465例初治MM（NDMM）患者的临床数据，比较了mSMART 3.0和mSMART 4.0分层体系下标危细胞遗传学异常及HRCA患者的临床特征及预后，研究HRCA对预后的影响。

## 病例与方法

1. 病例：纳入南京医科大学第一附属医院血液科2016年12月至2024年12月收治的465例NDMM患者。诊断符合《中国多发性骨髓瘤诊治指南（2024年修订）》的诊断标准[1]。

2. 细胞遗传学检测：采用胞质轻链免疫荧光结合荧光原位杂交（cIg-FISH）技术检测细胞遗传学异常。收集初治患者的骨髓液并分离单个核细胞，利用VYSIS双色标记探针标记检测位点，检测位点包括：17p缺失、1q21获得/扩增、1p32缺失、t（4;14）、t（11;14）、t（14;16）、t（14;20）。17p缺失和1号染色体异常（1q21获得/扩增、1p32缺失）的阳性阈值为20％，IgH重排的阳性阈值为10％[2]。运用二代测序（NGS）技术检测TP53基因是否发生突变。

3. 循环浆细胞检测方法及阈值设定：本中心采用多参数流式细胞术检测循环浆细胞水平，抽取患者外周血，共收集1×10^6^个细胞，采用单管8色方案检测CD38、CD138、CD45、CD19、CD56、CD27、cκ和cλ的表达，克隆性浆细胞CD19表达阴性，CD138、CD38、CD56表达增强，CD45、CD27表达减弱，限制性表达cκ或cλ。本文循环浆细胞的阈值设定采用本中心前期已发表的研究结果[3]–[4]，选取0.105％为循环浆细胞的截断值。

4. mSMART 3.0和mSMART 4.0分层系统对HRCA的定义[5]：mSMART 3.0的HRCA包括t（4;14）、t（14;16）、t（14;20）、17p缺失、TP53突变、1q获得。mSMART 4.0的HRCA包括：17p缺失和（或）TP53突变、1p双等位基因缺失、t（4;14）、t（14;16）、t（14;20）中任一种与1q获得/扩增或1p缺失同时出现，1q获得/扩增和1p缺失同时出现。

5. 随访：通过查阅住院或门诊病历以及电话联系的方式进行随访，随访截至2024年12月15日。无进展生存（PFS）期定义为患者自确诊至疾病进展或末次随访的时间，总生存（OS）期定义为患者自确诊至死亡或末次随访的时间。

6. 统计学处理：采用SPSS 26.0软件进行数据分析，采用GraphPad Prism 10.0软件作图。分类变量以例数（％）的形式描述，采用卡方检验进行组间比较。连续变量以*M*（范围）的形式描述。采用Kaplan-Meier法绘制生存曲线。应用Log-rank法比较组间生存差异。采用双侧检验统计数据，*P*<0.05为差异有统计学意义。

## 结果

1. 临床特征：465例NDMM患者中，男263例（56.6％），女202例（43.4％），中位确诊年龄为63（29～86）岁。根据mSMART 3.0风险分层系统，细胞遗传学标危（标危组）、高危（高危组）患者分别有161例（34.6％）、304例（65.4％）；根据mSMART 4.0风险分层系统，标危组、高危组患者分别有340例（73.1％）、125例（26.9％）。在mSMART 3.0和4.0分层系统中，标危、高危组患者的年龄、性别、M蛋白种类、乳酸脱氢酶、肌酐的差异均无统计学意义（*P*值均>0.05）。在两种分层系统中，高危组患者较标危组有更高的骨髓浆细胞及循环浆细胞比例和更低的PLT（*P*值均<0.05）。mSMART 4.0体系中HRCA患者发生贫血的比例高于标危组（*P*＝0.037）。mSMART 3.0体系中HRCA患者发生1q获得/扩增、17p缺失、t（4;14）的比例高于标危组（*P*值均<0.001），而t（11;14）比例低于标危组（*P*＝0.001）；在mSMART 4.0中，HRCA患者发生del（1p32）、1q获得/扩增、17p缺失、t（4;14）、t（14;16）、t（14;20）的比例高于标危组（*P*值均<0.001），而t（11;14）比例低于标危组（*P*＝0.002）。对两种分层系统中患者的治疗方案进行比较，标危、高危组患者治疗方案的差异均无统计学意义（*P*值均>0.05）。

2. 生存分析：中位随访37（1～96）个月。mSMART 3.0分层系统中180例HRCA患者在mSMART 4.0分层系统中被定义为标危，1例标危患者在mSMART 4.0分层系统中被定义为高危。在mSMART 3.0分层系统下，标危、高危组患者的中位PFS期分别为未达到和28（95％*CI*：23.2～32.8）个月（*P*<0.001），中位OS期分别为未达到和未达到（*P*<0.001）。在mSMART 4.0分层系统下，标危、高危组患者的中位PFS期分别为43（95％*CI*：36.4～49.6）个月和24（95％*CI*：15.7～32.3）个月（*P*<0.001），中位OS期均未达到（*P*<0.001）。结果提示，两种分层系统下，高危组患者的PFS期和OS期均较标危组缩短（图1）。

**图1:**
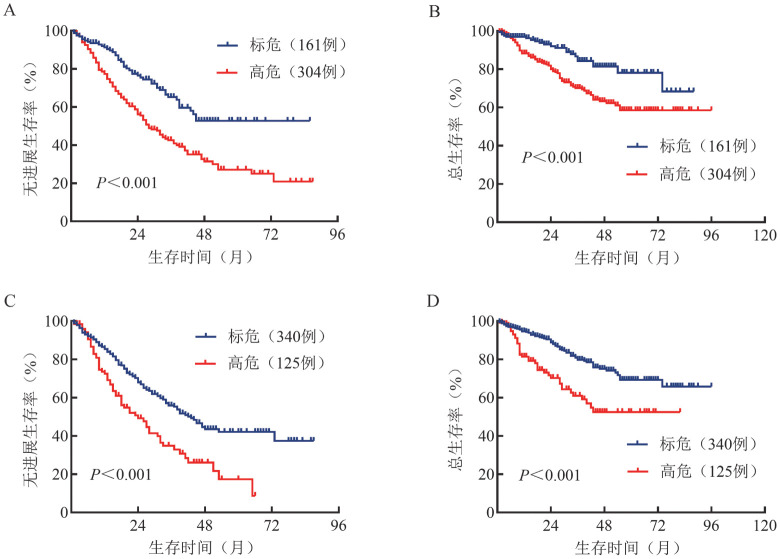
mSMART 3.0、mSMART 4.0分层体系下细胞遗传学标危与高危多发性骨髓瘤患者的无进展生存和总生存曲线 **注** mSMART 3.0分层体系下标危及高危患者的无进展生存（A）和总生存（B）曲线；mSMART 4.0分层体系下标危及高危患者的无进展生存（C）和总生存（D）曲线；mSMART：Mayo骨髓瘤分层及风险调整治疗

3. 伴多种细胞遗传学异常对预后的影响：在mSMART 3.0分层系统下，无HRCA、携带1种HRCA、2种HRCA、≥3种HRCA患者分别有161例（34.6％）、220例（47.3％）、69例（14.8％）、15例（3.2％），中位PFS期分别为未达到、29（95％*CI*：22.0～36.0）个月、28（95％*CI*：21.8～34.2）个月、18（95％*CI*：6.9～29.1）个月（*P*<0.001）；中位OS期分别为未达到、未达到、未达到和29（95％*CI*：17.1～41.0）个月（*P*<0.001）。值得关注的是，在mSMART 3.0分层系统下，携带1种HRCA和2种HRCA患者PFS（*P*＝0.805）及OS（*P*＝0.860）的差异均无统计学意义。在mSMART 4.0分层系统下，无患者携带≥3种HRCA，无HRCA、携带1种HRCA、2种HRCA患者分别有340例（73.1％）、104例（22.4％）、21例（4.5％），中位PFS期分别为43（95％*CI*：36.4～49.6）个月、28（95％*CI*：19.7～36.3）个月、10（95％*CI*：1.8～18.2）个月（*P*<0.001）；中位OS期分别为未达到、未达到、18（95％*CI*：5.7～30.3）个月（*P*<0.001），各亚组预后的差异均有统计学意义（*P*值均<0.001）（图2）。

**图2:**
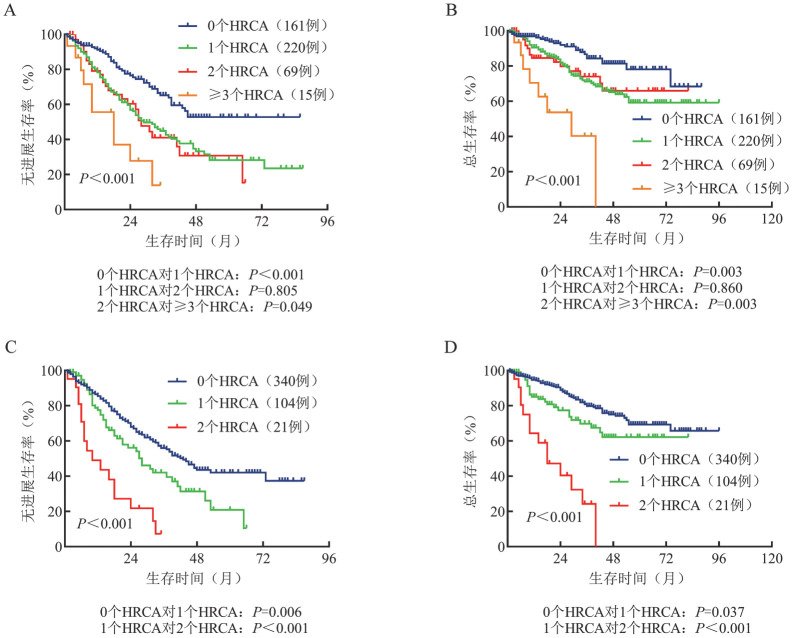
携带不同数量高危细胞遗传学异常（HRCA）多发性骨髓瘤患者的无进展生存和总生存曲线 **注** mSMART 3.0分层体系下，携带0、1、2、≥3个HRCA患者的无进展生存（A）及总生存（B）曲线；mSMART 4.0分层体系下，携带0、1、2个HRCA患者的无进展生存（C）及总生存（D）曲线；mSMART：Mayo骨髓瘤分层及风险调整治疗

## 讨论

细胞遗传学异常是MM危险分层体系中的核心危险因素，但HRCA的定义至今仍未达成一致。mSMART分层系统是美国梅奥诊所在大量临床研究的基础上，以细胞遗传学异常为主要指标建立的MM危险分层系统。2018年mSMART 3.0将t（4;14）、t（14;16）、t（14;20）、del（17p）、TP53突变、1q获得定义为HRCA。近日更新的mSMART 4.0分层系统对HRCA的定义有所调整。随着对MM遗传学认识的深化和新药的出现，HRCA的定义在不断演变，对MM的预后价值需要持续探索和评估。

本研究根据mSMART 3.0和mSMART 4.0对HRCA的定义，比较2个分层体系下标危细胞遗传学异常和HRCA患者临床特征及预后的差异。其中mSMART 4.0对HRCA的定义更为严格，也未提出“三打击”概念，或许是因为真实世界中这类患者较少，本中心也无患者携带≥3种mSMART 4.0定义下的HRCA。在这2种分层系统中，分别有65.4％和26.9％的NDMM患者被归为细胞遗传学高危，与标危组相比，其骨髓浆细胞及循环浆细胞比例更高，PLT更低，PFS及OS期明显缩短，提示遗传学高危患者肿瘤负荷高，造血功能抑制严重，预后更差。值得注意的是，mSMART 3.0对单打击、双打击患者的预后分层并不理想，调整后的mSMART 4.0分层更精准，可能是由于部分单个细胞遗传学异常并非独立的危险因素，高度相关的多个HRCA共存可能是预后差的主要原因。

del（17p）是MM中获得性细胞遗传学异常，发生率为5％～10％[6]，新药及自体造血干细胞移植仍难以克服其导致的不良结局[7]。mSMART 4.0将del（17p）阈值设定为20％，解决了既往研究中界值不一致的问题。TP53突变在MM中并不常见，发生率仅为3％～6％[8]，但其对MM预后影响较大。一项研究显示，在286例NDMM中，TP53突变率为3％，而约35％的del（17p）阳性患者存在TP53突变，可能原因是TP53单等位基因突变常继发于del（17p）[9]。与TP53突变或del（17p）患者相比，同时伴TP53突变和del（17p）患者的PFS期和OS期更短，提示两者存在协同作用[10]。本研究队列中，同时伴del（17p）和TP53突变的患者有6例，3例在14个月内死亡，预后极差。

1q21扩增在MM细胞遗传学异常中最常见，本中心检出率为54.6％。An等[11]的研究发现，携带1q21扩增拷贝数≥3与<3的患者预后无差别，而另有研究发现≥3个拷贝数患者的预后更差[12]，1q21拷贝数是否能纳入危险分层系统仍需国内外大样本研究进一步证实。1q扩增较少单独发生，合并del（1p）的发生率约为27.8％，伴IGH重排率为72.2％[13]。本中心合并del（1p32）患者仅占8.3％，可能是由于本中心仅检测del（1p32），合并IGH重排占比67.2％。del（1p32）虽发生率较低，但其危险度明显高于其他1p位点缺失[14]。本中心前期结果提示del（1p32）是NDMM预后不佳的独立危险因素，1q21获得/扩增和del（1p32）并存患者的预后较仅有其中一项异常患者更差[15]。2024 V1版NCCN指南首次将del（1p32）纳入HRCA[16]，mSMART 4.0对其进一步细化为只有1p双等位基因缺失被定义为HRCA。但由于样本数量受限，本研究未对1p是否为双等位基因缺失进行进一步分析，有待后续进一步数据支持。

前期研究显示，t（4;14）、t（14;16）、t（14;20）在NDMM中的发生率分别为15％、4％、1％[17]–[19]，数据显示，仅有t（4;14）异常患者的预后与无细胞遗传学异常患者无显著差异，同时伴有1q21获得患者的预后差于无细胞遗传学异常患者[20]。本研究中有81例患者伴t（4;14）异常，其中46.4％患者同时伴有1号染色体异常，仅有t（4;14）异常患者占14.8％。t（14;16）常伴随其他HRCA，分别有超过60％和20％患者同时伴有1q扩增和del（1p），其能否作为预后独立危险因素仍需探索[21]。本中心数据提示，携带t（14;16）异常的患者有5例，均伴1号染色体异常。综上，t（4;14）、t（14;16）、t（14;20）较少单独存在，独立存在的高危IGH易位在新药时代失去了对NDMM患者的预后评估价值，与其他遗传学异常同时存在时才能更准确地预测患者的预后。

本研究通过比较mSMART 3.0、mSMART 4.0分层体系对HRCA的定义比较不同细胞遗传学异常的预后评估价值。mSMART 4.0对HRCA的定义考虑了多种细胞遗传学异常同时出现的情况，对高危的标准较为严格。本研究支持mSMART 4.0对中国MM患者HRCA的定义，但鉴于本研究为单中心、回顾性研究，尚需多中心数据进一步证实。

## References

[1] 中国医师协会血液科医师分会, 中华医学会血液学分会 (2024). 中国多发性骨髓瘤诊治指南(2024年修订)[J]. 中华内科杂志.

[2] Ross FM, Avet-Loiseau H, Ameye G (2012). Report from the European Myeloma Network on interphase FISH in multiple myeloma and related disorders[J]. Haematologica.

[3] Han W, Jin Y, Xu M (2021). Prognostic value of circulating clonal plasma cells in newly diagnosed multiple myeloma[J]. Hematology.

[4] Xia Y, Shen N, Zhang R (2023). High-risk multiple myeloma predicted by circulating plasma cells and its genetic characteristics[J]. Front Oncol.

[5] MAYO CLINIC mSMART 4.0: Classification of active MM[EB/OL].

[6] Lannes R, Samur M, Perrot A (2023). In Multiple Myeloma, High-Risk Secondary Genetic Events Observed at Relapse Are Present From Diagnosis in Tiny, Undetectable Subclonal Populations[J]. J Clin Oncol.

[7] Marcoux C, Pasvolsky O, Milton DR (2025). Real-world outcomes of upfront autologous hematopoietic stem cell transplantation in patients with newly diagnosed multiple myeloma with deletion 17p[J]. Transplant Cell Ther.

[8] Lionetti M, Barbieri M, Manzoni M (2016). Molecular spectrum of TP53 mutations in plasma cell dyscrasias by next generation sequencing: an Italian cohort study and overview of the literature[J]. Oncotarget.

[9] Chin M, Sive JI, Allen C (2017). Prevalence and timing of TP53 mutations in del(17p) myeloma and effect on survival[J]. Blood Cancer J.

[10] Thanendrarajan S, Tian E, Qu P (2017). The level of deletion 17p and bi-allelic inactivation of TP53 has a significant impact on clinical outcome in multiple myeloma[J]. Haematologica.

[11] An G, Xu Y, Shi L (2014). Chromosome 1q21 gains confer inferior outcomes in multiple myeloma treated with bortezomib but copy number variation and percentage of plasma cells involved have no additional prognostic value[J]. Haematologica.

[12] Pasvolsky O, Ghanem S, Milton DR (2024). Outcomes of patients with multiple myeloma and 1q gain/amplification receiving autologous hematopoietic stem cell transplant: the MD Anderson cancer center experience[J]. Blood Cancer J.

[13] 刘 雪莲, 杨 珮钰, 于 小源 (2018). 1q21扩增对硼替佐米治疗初治多发性骨髓瘤患者疗效和预后的影响[J]. 中华血液学杂志.

[14] Li F, Hu L, Xu Y (2016). Identification of characteristic and prognostic values of chromosome 1p abnormality by multi-gene fluorescence in situ hybridization in multiple myeloma[J]. Leukemia.

[15] 郭 睿, 沈 旭星, 夏 园 (2024). 1p32缺失在初诊多发性骨髓瘤患者中的预后意义[J]. 中国实验血液学杂志.

[16] Kumar SK, Callander NS, Adekola K (2023). Multiple myeloma, version 2.2024, NCCN clinical practice guidelines in oncology[J]. J Natl Compr Canc Netw.

[17] Stong N, Ortiz-Estévez M, Towfic F (2023). The location of the t(4;14) translocation breakpoint within the NSD2 gene identifies a subset of patients with high-risk NDMM[J]. Blood.

[18] Mian H, Kaiser M, Fonseca R (2024). Still high risk? A review of translocation t(14;16) in multiple myeloma[J]. Am J Hematol.

[19] Vekemans MC, Lemmens H, Delforge M (2010). The t(14;20)(q32;q12): a rare cytogenetic change in multiple myeloma associated with poor outcome[J]. Br J Haematol.

[20] Geng C, Yang G, Zhou H (2023). Prognostic value of t(4;14) translocation in newly diagnosed multiple myeloma patients in novel agent era[J]. Hematology.

[21] Schavgoulidze A, Perrot A, Cazaubiel T (2023). Prognostic impact of translocation t(14;16) in multiple myeloma according to the presence of additional genetic lesions[J]. Blood Cancer J.

